# Comparison of the gut microbiota composition between wild and captive sika deer (*Cervus nippon hortulorum*) from feces by high-throughput sequencing

**DOI:** 10.1186/s13568-017-0517-8

**Published:** 2017-11-23

**Authors:** Yu Guan, Haitao Yang, Siyu Han, Limin Feng, Tianming Wang, Jianping Ge

**Affiliations:** Ministry of Education Key Laboratory for Biodiversity Science and Engineering and College of Life Sciences, Beijing Normal University, No. 19, Xinjiekouwai Street, Haidian District, Beijing, 100875 People’s Republic of China

**Keywords:** Sika deer (*Cervus nippon hortulorum*), Gut microbiota, 16s rRNA gene, High-throughput sequencing

## Abstract

The gut microbiota is characterized as a complex ecosystem that has effects on health and diseases of host with the interactions of many other factors together. Sika deer is the national level for the protection of wild animals in China. The available sequencing data of gut microbiota from feces of wild sika deer, especially for *Cervus nippon hortulorum* in Northeast China, are limited. Here, we characterized the gastrointestinal bacterial communities of wild (7 samples) and captive (12 samples) sika deer from feces, and compared their gut microbiota by analyzing the V3–V4 region of 16S rRNA gene using high-throughput sequencing technology on the Illumina Hiseq platform. *Firmicutes* (77.624%), *Bacteroidetes* (18.288%) and *Tenericutes* (1.342%) were the most predominant phyla in wild sika deer. While in captive sika deer, *Firmicutes* (50.710%) was the dominant phylum, followed by *Bacteroidetes* (31.996%) and *Proteobacteria* (4.806%). A total of 9 major phyla, 22 families and 30 genera among gastrointestinal bacterial communities showed significant differences between wild and captive sika deer. The specific function and mechanism of *Tenericutes* in wild sika deer need further study. Our results indicated that captive sika deer in farm had higher fecal bacterial diversity than the wild. Abundance and quantity of diet source for sika deer played crucial role in shaping the composition and structure of gut microbiota.

**Electronic supplementary material:**

The online version of this article (10.1186/s13568-017-0517-8) contains supplementary material, which is available to authorized users.

## Introduction

The sika deer (*Cervus nippon*) is a cervidae species, which distributed extensively in East Asia, including China, Korea, Vietnam and Taiwan, especially the Japanese archipelago (Goodman et al. 2001). A total of six subspecies of sika deer distributed in China historically but there are only three subspecies remaining now in mainland China: *C.n. hortulorum*, *C.n. sichuanicus* and *C.n. kopschi* (McCullough et al. 2009). Although listed as least concern (LC) class by international union for conservation of nature (IUCN) on account of abundant and increasing populations in Japan and Russia (Harris 2015), sika deer was listed as the first-grade state protection animal and Endangered class in China. The number of wild sika deer (*C.n. hortulorum*) in Northeast China was less than 500 according to the relevant wild resources survey by country in 1995 due to over-hunting, habitat loss and fragmentation. While the domesticated sika deer are approximately 550,000 heads now in China (Li et al. 2013). Moreover, overwhelming majority of sika deer mainly inhabit in Hunchun, Dunhua, Antu and Fusong of Jilin province. Meanwhile, as the momentous prey of Amur tiger (*Panthera tigris altaica*) in the coniferous and broadleaved mixed forests in Northeast China, sika deer also plays important role in affecting the distribution and abundance of the former (Miquelle et al. 1996), as well as even in maintaining the ecosystem balance (Huang et al. 2015). Sika deer also is famous as its economic and medical value in traditional Chinese medicine, such as velvet antlers, meat and skin, in particular the wild sika deer, as well as its auspicious sign since ancient times.

Albeit part of researches have done for species diversity of sika deer on the basis of molecular biology in China (Lü et al. 2006; Liu et al. 2002; Wang et al. 2008; Wu et al. 2004, 2005), most of the previous studies aimed to its distribution, amount, activity patterns and trophic analysis (Fu et al. 2006; Huang et al. 2015; Liu et al. 1999; Lui et al. 2003; Xiao et al. 2014). Gut microbiota, as a prevalent and rapid-developed method owe to the next sequencing technology, becomes more and more vital for comprehending developmental, immunological and physiological functions which affects health and diseases of both human and wild animals (Drasar and Hill 1974; Guarner and Malagelada 2003; Nicholson et al. 2005). However, the detailed data, especially the gut microbiota data of wild sika deer in Northeast China, are comparatively insufficient. Given that the plight of protection for wild sika deer in Northeast China, our prime aim of this work is to characterize the basic fecal bacterial community composition and structure of wild sika deer, and then make a comparison of gut microbiota between wild and captive sika deer living in distinct environment, which may make a contribution to the study of gut microbiota for wild animals and provide ideas for protection toward these endangered and precious cervidae species.

## Materials and methods

### Samples collection

A total of 19 fresh feces samples of sika deer were all collected during February and March 2017. According to the size of home range of sika deer, feces samples of wild sika deer (W1–W7) were collected from seven sites (Table 1) in Hunchun, Jilin province of China, which were designed severely for collection with sufficient distance separation, within 3 days after a heavy snow to ensure that each feces sample belonged to different sika deer individual. The temperature lower than 0 °C in Hunchun kept the feces of wild sika deer fresh as much as possible.

**Table 1:** Information of feces samples from sika deer

Group	Sample name	Sampling site	Coordinates	
			Latitude	Longitude
Wild	W1	Hunchun	42.8748	130.9424
	W2	Hunchun	43.0783	131.1399
	W3	Hunchun	42.6865	130.4513
	W4	Hunchun	43.0177	130.8364
	W5	Hunchun	43.1214	130.6774
	W6	Hunchun	42.8330	130.5497
	W7	Hunchun	42.8875	130.7625
Captive	C1–C12	Beijing	39.8586	116.7755

Feces samples from captive sika deer (C1–C12) were collected from Beijing Nine Deer Industry Co., Ltd. To keep the feces fresh, the barns of sika deer were cleaned in advance and all feces were collected immediately within a ½ h after defection. All the captive sika deer in deer farm were healthy and not injected any antibiotics or antiphlogistic drugs during the past 3 months.

All the fecal samples were stored in the dedicated chest freezer and then frozen at − 80 °C for further experiments.

### DNA extraction

According to the instruction of kit, total genome DNA from fecal samples was extracted using QIAamp^®^ Stool Mini Kit (Qiagen, Germany).

### 16S rRNA gene PCR and sequencing

16S rRNA gene was amplified using the 16S universal amplicon PCR primers: forward primer (CTACGGGNGGCWGCAG) and reverse primer (GACTACHVGGGTATCTAATCC), and V3–V4 region of 16S rRNA gene were our final target fragments for sequencing. A total final volume of 50 μL mixture for polymerase chain reaction: 6 μL of template fecal DNA, 25 μL of 2 × Taq PCR Master Mix (0.1 U/μL), 2 μL of each primer (10 μM) and 15 μL ddH_2_O to complement the reaction system. Then DNA was amplified using the conditions below: 3 min at 95 °C for initial denaturation, then followed 25 cycles of 95 °C for 30 s, 55 °C for 30 s and 72 °C for 30 s. Finally, followed by an extension step of 72 °C for 5 min.

The PCR products were mixed with SyBR^®^ Safe DNA Gel Stain (Invitrogen, USA) and estimated on 2% agarose gel by electrophoresis. Then PCR products were purified with Qiagen Gel Extraction Kit (Qiagen, Germany) for library preparation and sequencing. TruSeq^®^ DNA PCR-Free Sample Preparation Kit (Illumina, USA) were used to generate the sequencing libraries. And then, sequencing for mixed PCR products were conducted on an Illumina Hiseq 2500 platform following the manufacturer’s recommendations.

### Sequence processing and analysis

The effective tags obtained after paired-end reads assembly and quality control from original data. The sequence assembly and quality filtering on the raw tags were performed using FLASH (Magoč and Salzberg 2011) and QIIME (Version 1.7.0) software (Bokulich et al. 2013) respectively. To remove the chimeric sequences, reference database was compared with the tags we obtained using UCHIME algorithm (Edgar et al. 2011). Then we assigned sequences into the same operational taxonomic unit (OTU) with the similarity greater than or equal to 97% and made species annotation based on RDP classifier algorithm (Wang et al. 2007) using the GreenGene Database (DeSantis et al. 2006).

All of the indices of Alpha diversity, including Chao, ACE, Shannon, Simpson, Coverage, and the Beta diversity analysis for species complexity in our fecal samples were calculated with QIIME (Caporaso et al. 2010). The Rarefaction Curves and Rank Abundance Curves were displayed with R software, as well as the clustered heatmap at genus level. T test was used to analyze the discrepancies between wild and captive samples for both indices of Alpha and Beta diversity. We used Analysis of Similarities (ANOSIM) to test statistically whether there is a significant difference between two groups (Clarke 1993). R software was also applied to calculate and display the T test bar plot, principal component analysis (PCA), principal co-ordinate analysis (PCoA) and non-metric multidimensional scaling (NMDS). Unweighted pair-group method with arithmetic means (UPGMA) to evaluate the similarity and discrepancies of fecal bacterial community among samples based on weighted and unweighted distance matrix at different levels. Linear discriminant analysis coupled with effect size (LEfse) was generated by LEfse software and the filter value of LDA score was set as 4 by default (Segata et al. 2011).

The obtained data available for our study were submitted to NCBI sequence read archive (SRA) under the study Accession Number: SRP107844.

## Results

### Overview of the sequencing data

**Fig. 1:**
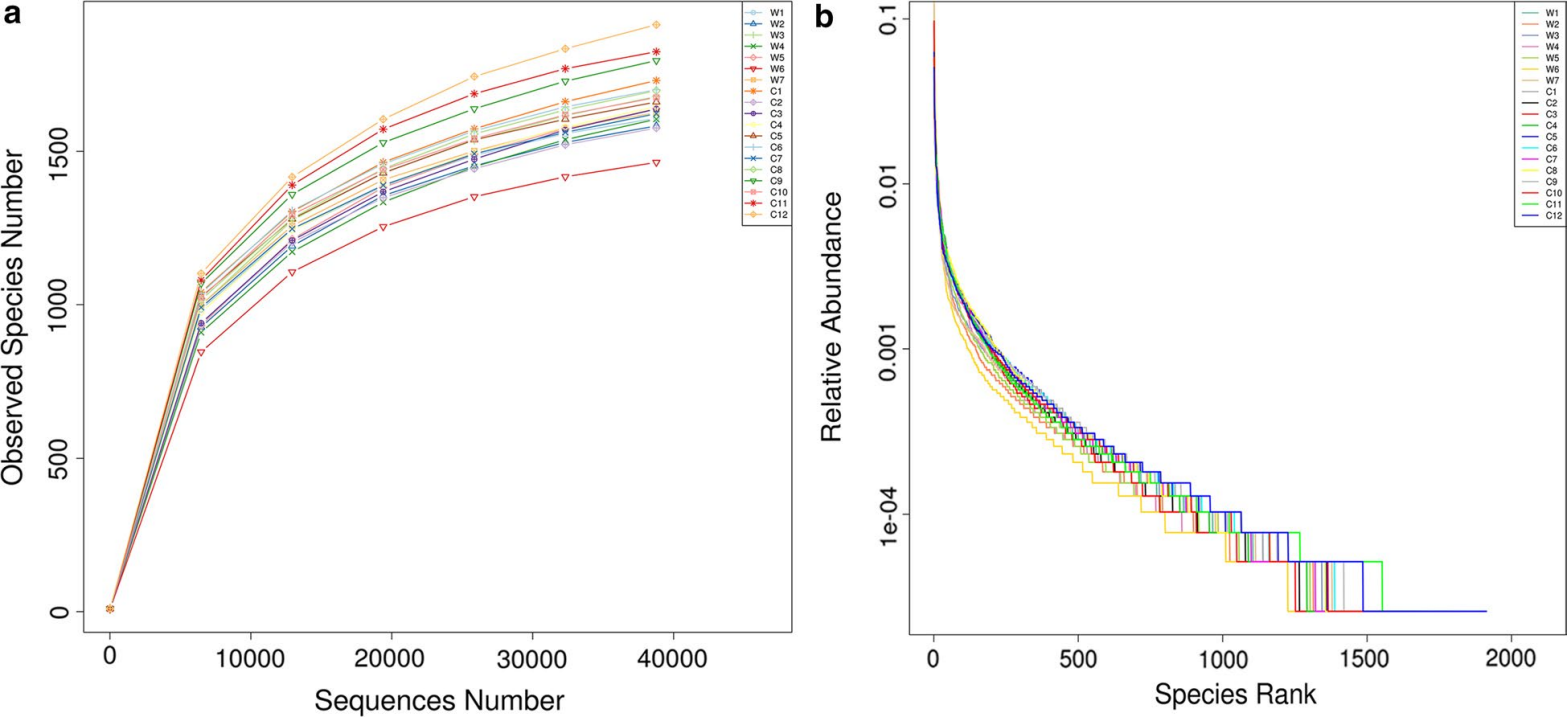
Rarefaction curves (**a**) and rank abundance curves (**b**). The former reflects the rationality of our sequencing data size and abundance of species in feces samples indirectly. In rank abundance curves, wider span of curves reveals higher relative abundance of species in horizontal direction and the smoothness of curves showed the evenness of bacterial species in samples vertically

A total of 1,383,782 high quality reads were obtained after the quality control and classified into 3059 OTUs with the 97% similarity from 19 feces samples of sika deer. The reads we used for the next bacterial community diversity analyses and the Alpha-diversity indices (including observed species, Shannon, Chao1, ACE and Good coverage) were shown in Table 2. The rarefaction curves (Fig. 1a) became flat gradually and almost got a plateau with more data indicated that the number of OTUs we analyzed for each sample were sufficient and reasonable. And also the rank abundance curves that reflected the evenness and abundance of species in fecal samples horizontally and vertically were demonstrated in Fig. 1b.

**Table 2:** Alpha-diversity of gut microbiota in feces samples from wild and captive sika Deer

Sample	Observed species	Shannon	Simpson	Chao1	ACE	Good coverage
W1	1608	8.335	0.986	1734.500	1767.797	0.993
W2	1583	7.678	0.973	1708.224	1765.752	0.993
W3	1679	8.155	0.981	1953.537	1931.340	0.991
W4	1605	8.091	0.986	1938.380	1939.482	0.990
W5	1639	7.861	0.977	1868.215	1881.464	0.991
W6	1464	7.068	0.961	1595.671	1607.925	0.994
W7	1626	7.902	0.972	1741.143	1784.583	0.994
C1	1731	8.742	0.993	2048.050	2036.769	0.990
C2	1576	8.352	0.989	1832.410	1799.409	0.992
C3	1638	8.178	0.984	2004.132	1965.689	0.990
C4	1646	8.521	0.992	1954.015	1928.890	0.991
C5	1661	8.730	0.992	1926.588	1866.773	0.992
C6	1701	8.709	0.993	1916.531	1922.444	0.992
C7	1623	8.550	0.991	1828.170	1842.684	0.992
C8	1698	8.796	0.994	2001.902	1962.328	0.991
C9	1796	8.859	0.994	2100.189	2085.281	0.990
C10	1676	8.576	0.991	1914.995	1889.268	0.992
C11	1825	8.631	0.992	1954.818	1986.782	0.993
C12	1913	8.741	0.992	2264.454	2255.763	0.989

### Bacteria composition and relative abundance

We totally detected 29 phyla, 60 classes, 104 orders, 179 families and 345 genera in the gut microbiota community from these 19 feces samples of sika deer.

At phylum level of wild sika deer, *Firmicutes* (77.624%) was the predominant phylum, followed by *Bacteroidetes* (18.288%) and *Tenericutes* (1.342%). Then is *Proteobacteria* (0.540%). What is noteworthy is that *Actinobacteria* (0.411%) was not the most dominant phylum whose rank of relative abundance should be one of the top three or five as usual. While *Ruminococcaceae_UCG*-*005*, *Ruminococcaceae_UCG*-*010* and *Christensenellaceae_R*-*7_group* were the prevalent genera in wild sika deer group which all belonged to *Firmicutes*.

**Fig. 2:**
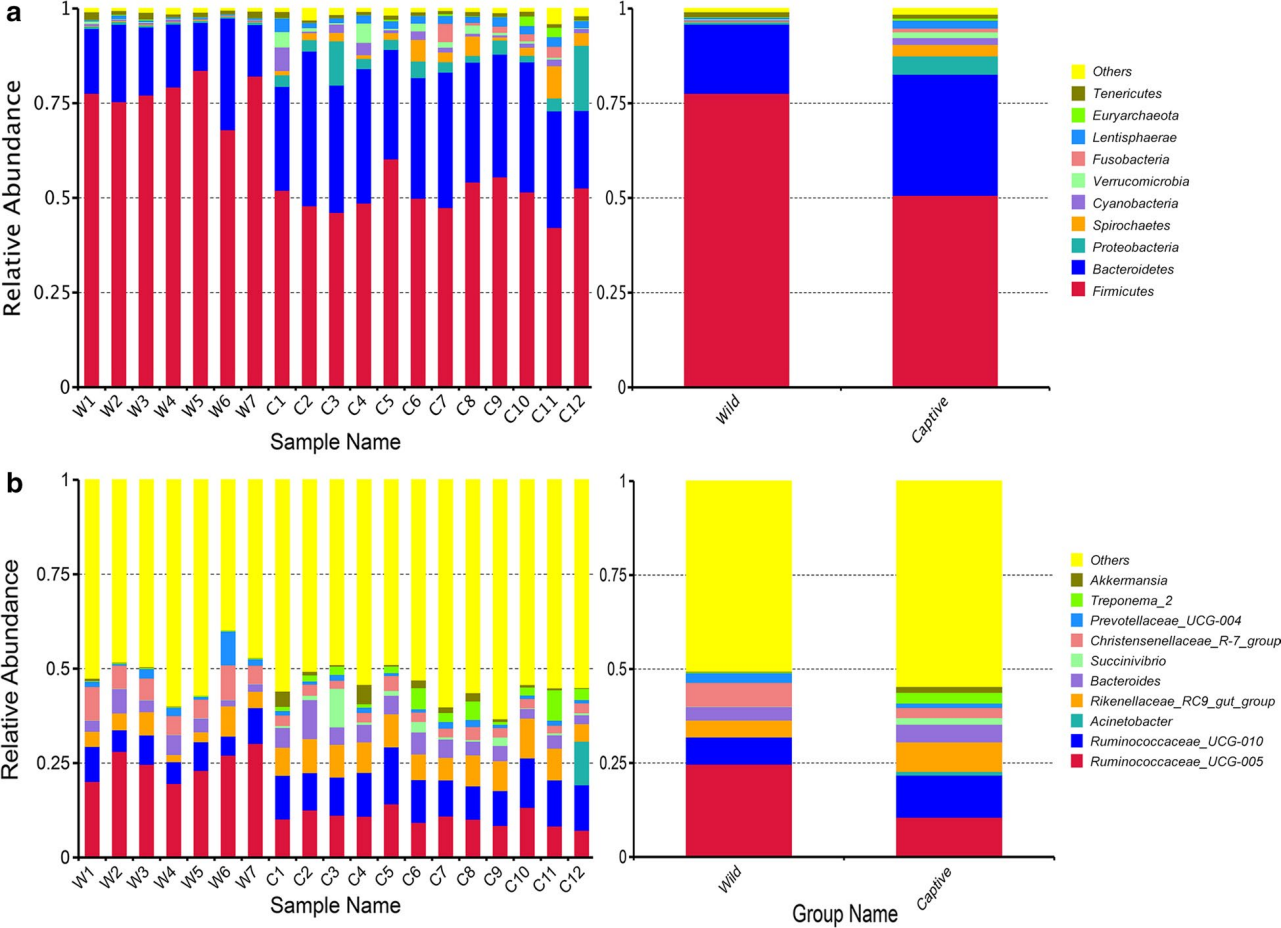
Fecal microbial composition of wild and captive sika deer at the phylum (**a**) and genus (**b**) level. Each bar represents the top ten bacterial species ranked by the relative abundance in each individual sample or group

For captive sika deer, *Firmicutes* (50.710%) and *Bacteroidetes* (31.996%) were also the dominant phyla as wild group, then followed by *Proteobacteria* (4.806%). And at the genus level, *Ruminococcaceae_UCG*-*005*, *Ruminococcaceae_UCG*-*010* and *Rikenellaceae_RC9_gut_group* (belonged to *Spirochaetes*) were the most common genera. To show the relative abundance of bacterial communities more intuitively, we chosen the top 10 species for each sample or group and generated the percentage stacked histogram of relative abundance at phylum and genus level in Fig. 2a, b respectively.

**Fig. 3:**
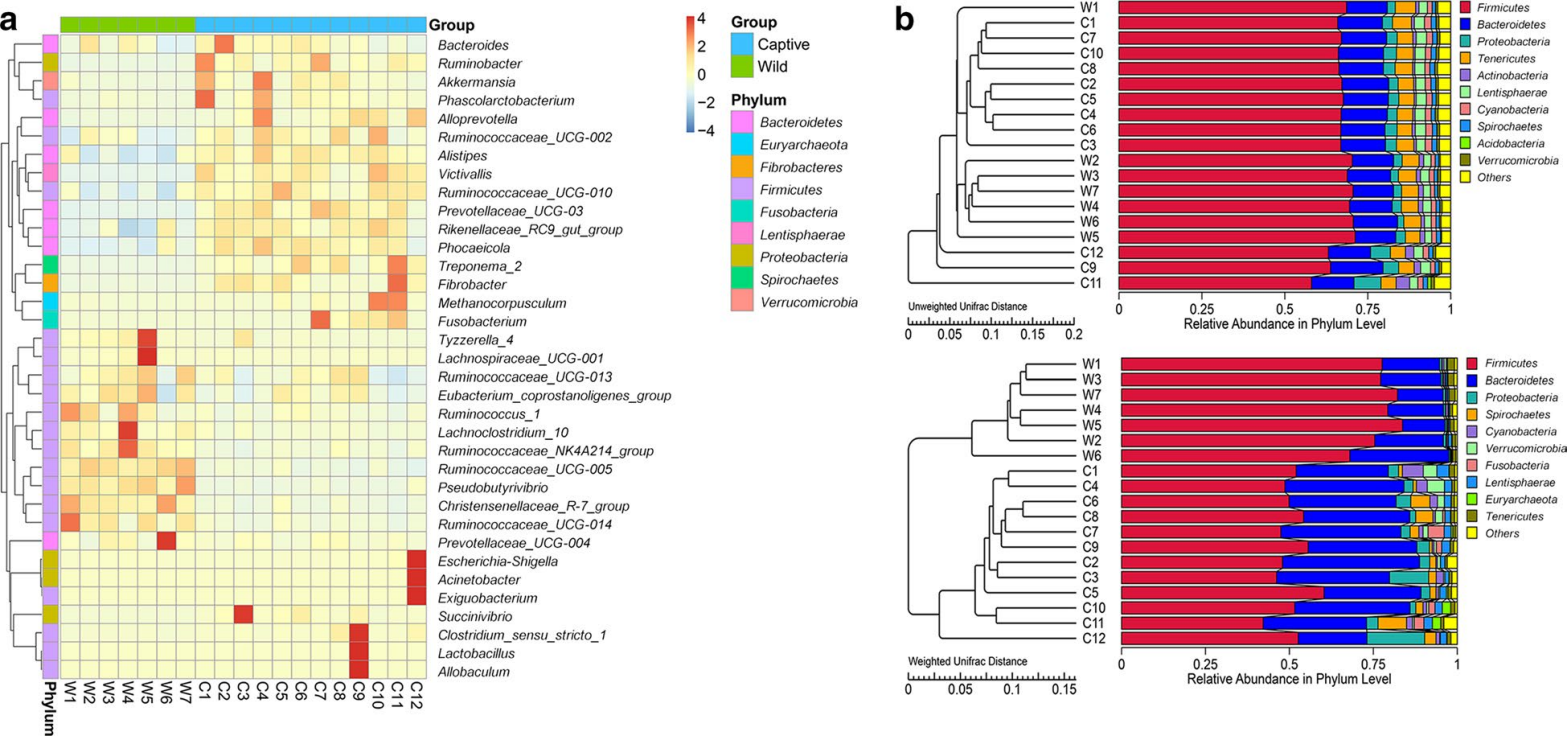
The heatmap of clustering for species abundance (**a**). The information of samples and species annotation were demonstrated along *X*-axis and *Y*-axis respectively. The clustering tree was generated based on the relative abundance of the genera in the top 35. The relative values in heatmap depicted by colors after normalization indicated the aggregation degree or content of bacterial species among samples at genus level. UPMGA clustering trees (**b**)—based on unweighted Unifrac distance and weighted Unifrac distance. The results of clustering using two distance matrixes were combined with the overall percentages of relative abundance among all samples at phylum level

The heatmap for clustering with relative abundance of species at genus level was demonstrated in Fig. 3a. According to the clustered heatmap, wild sika deer (W1–W7) were grouped together while captive sika deer (C1–C12) were grouped in the other one. The genera that accounted for different proportion were also presented by different colors and locations of clustering in heatmap. With the weighted Unifrac and unweighted Unifrac distance matrix, we made the unweighted pair-group method with arithmetic mean (UPGMA) clustering analysis to study the similarity between samples in Fig. 3b. The dendrograms of UPGMA was similar to the result in clustered heatmap.

### Analysis of discrepancies for between-group

**Fig. 4:**
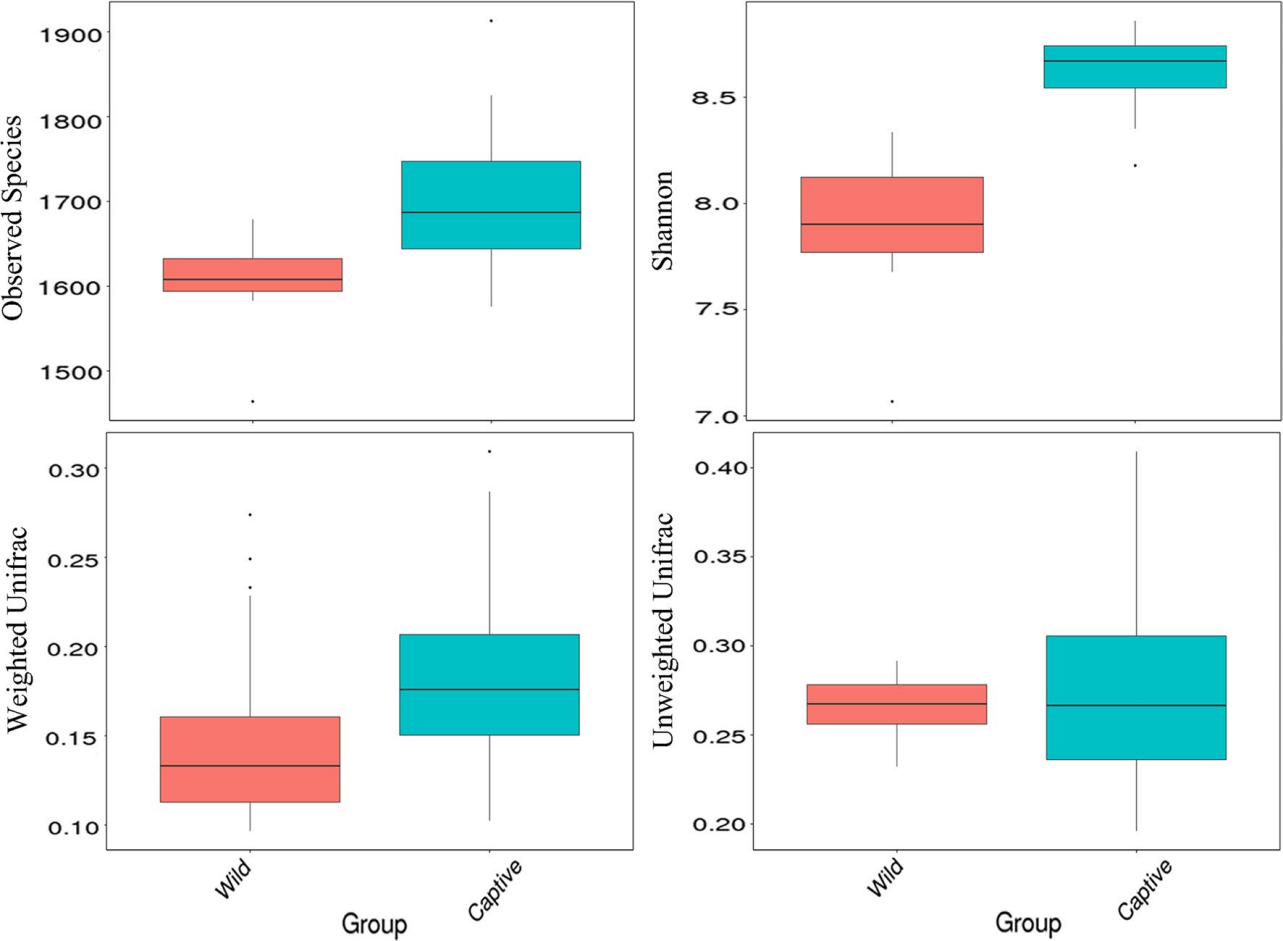
The comparisons for Alpha-diversity (observed species and Shannon index) and beta-diversity (with weighted and unweighted Unifrac distance matrix) between wild and captive sika deer

**Fig. 5:**
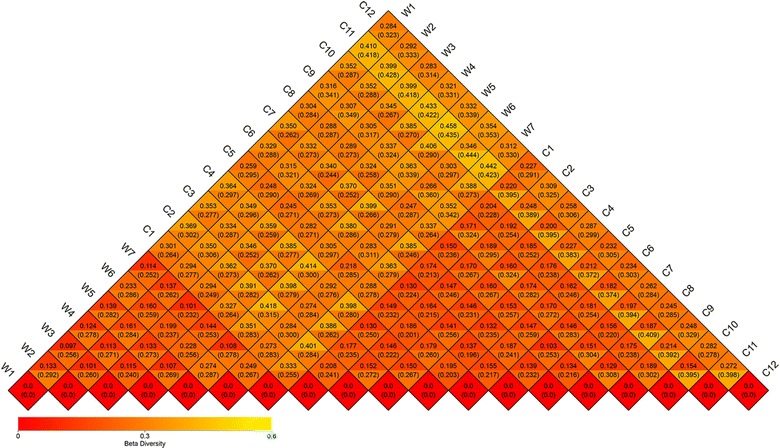
Heatmap of beta-diversity. The numbers in grids are the dissimilarity coefficient between samples. The diversity of bacterial species is proportional to the dissimilarity coefficient. And the two numbers in the same grid represent weighted and unweighted Unifrac distance respectively

**Fig. 6:**
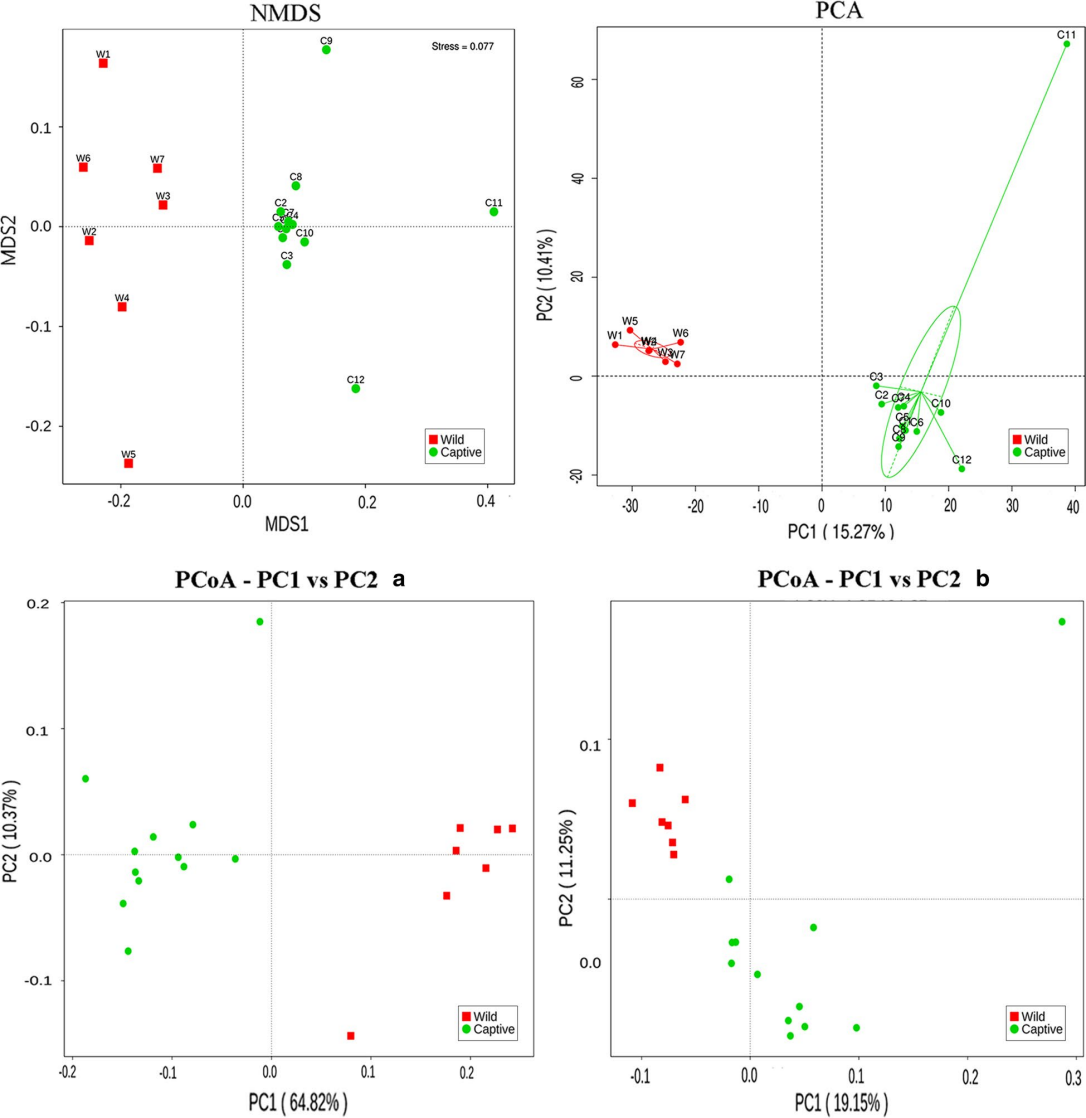
NMDS, PCA and PCoA of the bacterial population structures. The red and green dots represented wild and captive sika deer samples respectively. For PCoA, (**a**) was generated with weighted Unifrac distance while (**b**) used unweighted Unifrac distance

The T test of Alpha (observed species and Shannon) and Beta-diversity (using Unweighted and Weighted Unifrac distance matrix) between wild and captive groups were shown in Fig. 4 (*P* = 0.012, 0.002, 0.056, 0.016). Then we used Analysis of Similarities (ANOSIM, Additional file 1: Figure S1) to test if the division of groups is reasonable (R = 0.998, *P* = 0.001) and the result supported our original design for grouping of sika deer. The heatmap of Beta-diversity index calculated by weighted Unifrac distance and unweighted Unifrac distance was plotted in Fig. 5 to suggest the discrepancy of species diversity between samples. To analyze the discrepancy between groups further, we also demonstrated the non-metric multi-dimensional scaling (NMDS) plot, the principle component analysis (PCA) plot and the principle co-ordinates analysis (PCoA) plots in Fig. 6. Among these plots, PCoA plots (Fig. 6a, b) were generated using the two distance matrixes mentioned above. The distance between the dots with two colors showed the similarity of their own bacterial community structure. Wild and captive sika deer tended to gather together within respective group obviously.

**Fig. 7:**
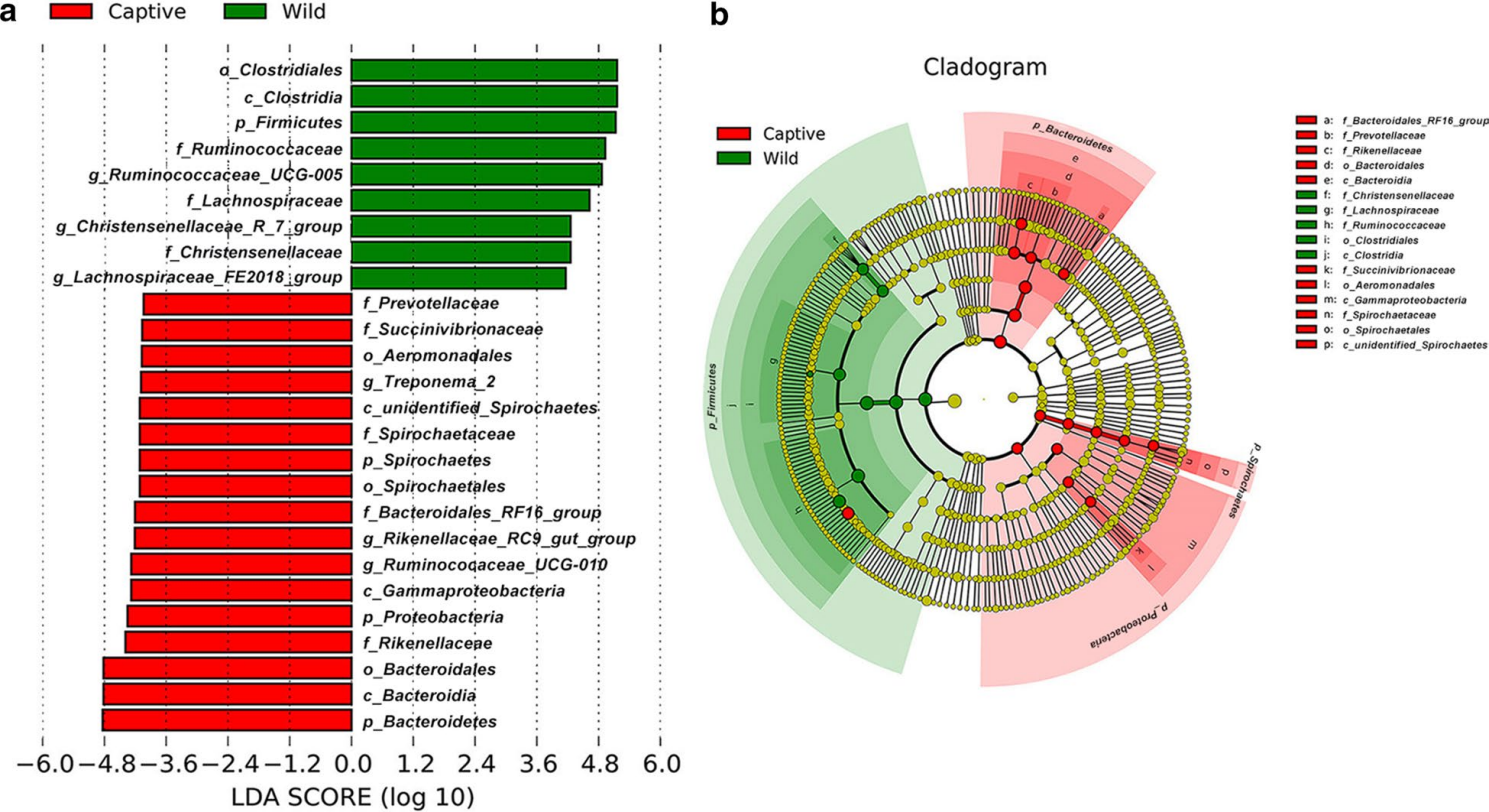
The results of LEfSe (LDA Effect Size) analysis (**a**). The histogram of LDA score showed the biomarkers with statistics difference between groups. The influencing degree of species was expressed by the length of bar in histogram. In the cladogram (**b**), the circle radiated inside-out demonstrated the classification (from phylum to genus). Each small circle at different classification represented a taxa and the diameter of circle is proportional to the relative abundance. The species not with significant differences were colored by yellow and biomarkers were colored by different groups. Red and green dots represent the core bacterial populations in respective group

The specific species that had significant difference between groups at each level was calculated using T test and LDA effect size (LEfSe) analyses. At phylum level, the relative abundance of *Firmicutes*, *Bacteroidetes* and *Lentisphaerae* showed remarkable difference (*P* < 0.001) in wild group. *Proteobacteria*, *Spirochaetes* and *Fibrobacteres* were significantly higher among captive sika deer (*P* = 0.009, 0.002, 0.004 respectively). Other species with significant discrepancy at phylum, family and genus level were presented in T test bar plot, as well as the relative abundance and *p* value in Additional file 2: Figure S2. The LEfSe analysis provided us the taxa that with significantly different abundance between wild and captive groups in Fig. 7a. A total of nine and seventeen taxa that had discrepancy in relative abundance were presented in wild and captive groups respectively (e.g. *Firmicutes*, *Aeromonadales*, *Ruminococcaceae* spp., *Spirochaetaceae*). The cladogram in Fig. 7b showed the core bacterial species with remarkable difference at all levels.

## Discussion

Along with the high-throughput sequencing technology more and more thorough and widespread application, as well as the deeper progress in the field of gut microbiota for mammals, the comprehensions for gastrointestinal microecology and health of wild animals have become increasingly clear to us.

Sika deer (*Cervus nippon hortulorum*) is listed as the first-grade state protection animal in China. While as the representative herbivorous animal and momentous prey of Amur tiger in Northeast China, previous researches are mostly focused on the captive or domestic sika deer individuals. The available high-throughput sequencing data of gut microbiota from wild sika deer are still limited so far, particularly for analysis of discrepancies between wild and captive sika deer populations.

We characterized the primary composition, structure and the comparison of intestinal microflora from feces between wild and captive sika deer in this study.

In general, the results our study demonstrated were consistent basically with the previous characterizations of intestinal bacterial community for herbivorous, like musk deer (Hu et al. 2017), horses (Costa et al. 2012) and cattle (Whitford et al. 1998).

As the most predominant phylum, the relative abundance of *Firmicutes* (77.624%) in wild sika deer was significantly different than captive group (50.710%, *P* < 0.001). However, *Firmicutes* might not always be the most predominant phylum that could be ranked after *Bacteroidetes* in some studies about carnivorous animals, such as dhole (Wu et al. 2016). While an obvious increase in *Firmicutes* when there was a food supplementation with fiber in dogs had been reported (Middelbos et al. 2010). Considering the dietary components of wild sika deer in winter were composing prevailingly of *Pinaceae* plants, *Salicaceae* plants, branches and leaves of *Quercus mongolica*, even the barks when food scarcity occurred (Tsujino and Yumoto 2004), the remarkable difference we found in this staple phylum between two groups might be related to high-fiber diet of wild sika deer for ingesting indigestible parts in food or fermentation in gastrointestinal tract (Costa et al. 2012).

However, although another phylum *Fibrobacteres* were all detected in these 19 feces samples, the relative abundance of *Fibrobacteres* (*P* = 0.004) was significantly higher in captive sika deer. Specifically, as the main cellulolytic bacteria, *Fibrobacter succinogenes* belonged to this phylum which is essential for fiber degradation in rumen (Jami and Mizrahi 2012). The detection of *Fibrobacteres* was connected to the content of fiber in different diet in previous researches, especially in animals those were fed in hay primarily (Fernando et al. 2010; Tajima et al. 2001). *Fibrobacteres succinogenes* also decreased while animals were fed with diet that contains high fiber content. This result indicated that captive sika deer may experience certain adaption gradually as the fiber content changed in diet. Thus, according to the different content of these two phyla about fiber degradation in two groups, although dietary plays an important role in changing composition and structure of intestinal bacterial communities, there are still lots of other key factors which could influence gut microbiota potentially, such as environmental and seasonal variations. Exactly, some previous studies indicated that ingested foods and ruminal characteristics of wild sika deer were distinct greatly as the seasons change in Japan (Ichimura et al. 2004; Yokoyama 1996).

*Bacteroidetes* is another dominant phylum among mammalian animals, which was significantly higher (*P* < 0.001) in captive sika deer (31.996%) compared with 18.288% in wild group. As a crucial phylum in gut microbiota, *Bacteroidetes* was functional for degradations of high molecular weight substances and carbohydrates that from intestinal secretions (Salyers et al. 1977; Thoetkiattikul et al. 2013). The high relative abundance of *Bacteroidetes* had been reported in children with high-fiber dietary from rural Africa (De Filippo et al. 2010). Similarly, the increase in *Bacteroidetes* was also observed as the weight lose of obese mice while not significantly correlated to total calorie intake (Ley et al. 2006; Turnbaugh et al. 2006). Furthermore, the higher relative abundance in *Bacteroidetes* and lower in *Firmicutes* of captive sika deer suggested that the dietary proportions in deer farms which were gathered with poplar leaves, corn stalks and peanut straw might not be truly heathy and may result in weight loss to some extent for captive sika deer. After all, the ultimate purposes of sika deer farms are various, including antlers, meat and blood, due to great economic values under the background of traditional Chinese medicine, and the authentic health conditions of sika deer cannot be measured by scientific methods, such as analyses for gut microbiota. Additionally, the *Firmicutes*/*Bacteroidetes* ratio also evolved during different stages of life in human gut microbiota that represented distinct diversity of bacterial communities and digestive ability (Mariat et al. 2009). However, some individual variations in bacterial community composition had been reported in similar ruminants, such as Canadian cervids (Gruninger et al. 2014). Due to the hardships of wild samples collection and the principles of non-invasive sampling, more specific studies and characterizations of these phyla in herbivorous animals should be conducted further in the future.

Interestingly, *Tenericutes* (1.342%) was one of the most predominant phyla in wild sika deer, which ranked before *Proteobacteria* (0.540%). Nevertheless, the result of comparison about this phylum indicated no obvious difference here between two groups of sika deer. Phylum *Tenericutes*, class *Mollicutes* were rarely found in gastrointestinal tract before and then were identified for the first time in wild chimpanzee (McLaughlin et al. 2012; Szekely et al. 2010). Moreover, *Tenericutes* were discovered in both carnivorous and herbivorous mammals extensively, such as sables and cows (Guan et al. 2016; Jami and Mizrahi 2012), and also in aquatic animals, like Yangtze finless porpoise (McLaughlin et al. 2012). Conversely, the sequences that belonged to *Tenericutes* were not detected in seals (Glad et al. 2010). Due to the presence of *Mollicutes* on both healthy and unhealthy humans, and its possible pathogenicity for hosts (Eckburg et al. 2005; Ley et al. 2006; Novy et al. 2009; Palmer et al. 2007; TaylorRobinson 1996; Turnbaugh et al. 2008), the higher relative abundance and function of *Tenericutes* in wild sika deer should be paid more attention.

Plus, the relative abundance of *Proteobacteria* was higher in captive sika (4.806%) deer than the wild and also had significant difference (*P* = 0.009) between groups. *Proteobacteria* was the most predominant phylum in giant panda which could help degrade lignin in its main food source (Fang et al. 2012). This phylum was also related to catabolize various components which from animal fodder in bovine (Evans et al. 2011). We inferred that higher relative abundance of *Proteobacteria* in captive sika deer was possibly due to the compositions of dietary, which was made artificially by deer farm, including certain nutrients or unknown stuff that cannot be digested easily. In contrast, the major components of dietary for wild sika deer seemed to be not comparatively stable. Interestingly, for storing more fat and surviving in long winter, sika deer would establish different foraging strategies due to the deficient approaches to good food resources, including more active rumen fermentation on fibrous diets (Ichimura et al. 2004; Yokoyama et al. 2000, 2001). Based on these results, we also inferred that wild sika deer has adopted themselves to the hostile environments constantly with the advantages of body size (Suzuki et al. 2001) and abilities that absorb nutrition from indigestible food diet in winter.

At the genus level, as the previous research of golden takin basically (Chen et al. 2017), *Ruminococcaceae_UCG*-*005* (*P* < 0.001) and *Ruminococcaceae_UCG*-*010* (*P* < 0.001) were the dominant and higher abundant genera in captive sika deer belonged to *Ruminococcaceae*, which could make contributions to fiber digestion. Another dominant genus that detected in rumen of domestic Sika deer in China and other ruminants (Qian et al. 2017) before was *Prevotella*, which may be related to the degradation of fiber biomass or tannins (Li et al. 2013). And *Prevotella bryantii* populations would increased multiply as the animals were adapted to the high-grain diet (Fernando et al. 2010). However, *Prevotella* was not the most dominant genus in our study in wild or captive sika deer. We suspected that the most possible reason was the samples source in which the compositions and abundance of gut microbiota varied throughout the gut (Mueller et al. 2012). Our samples were all from fresh feces while the samples mentioned in above study were from the contents of rumens in sika deer. Therefore, samples from each crucial part of gastrointestinal tract in sika deer need to be collected further and studied in more detail by following researches.

Taken together, major phyla and genera of bacteria related to fiber digestion and food fermentation were almost indicating that captive sika deer in our study had the more diverse bacterial communities and abundant sources of food.

Indeed, the test results for Alpha and Beta-diversity (Fig. 4) of species we identified from feces were overall consistent with the inference above, as well as the analyses for similarities between two sika deer groups in Additional file 1: Figure S1. And also the rarefaction curves (Fig. 1) displayed the coherent result. It’s worth nothing that the diversity of the intestinal bacteria communities of wild sika deer was lower than the captive, which was contrary to partial reports that wild animals contains more abundant and complex gut microbiota. Thus, we hypothesized that although having many advantages for captive sika deer in deer farm, such as the comparative steady living environment and high degree of freedom for activity, food source during long winter is still the most important constraint in altering the major structure of bacterial communities. Additionally, the NMDS, PCA and PCoA analyses using different methods were also showing the distinct separations and reciprocal relationships among sika deer from unlike environment. Wild and captive sika deer were clustered observably into two groups in both heatmap and phylogenetic trees, which further indicated that the composition and structure of bacterial community were significantly different between wild and captive group.

Moreover, age, sex and host genetics would also be non-negligible influencing factors for gut microbiota in mammals (Zhang et al. 2010). Given that the basic information and health conditions of wild sika deer were unknown due to the rigorous protection in China, more detail studies for captive sika deer should be conducted primarily to investigate the possible reasons of these changes in bacterial community of gastrointestinal tract. More wild feces samples should be collected and added to the analysis in both bacterial community and specific physiological parameters, such as back fat thickness and kidney fat index (KFI) (Riney 1955), which could combine those specific microbiome to behaviors and diseases further of sika deer.

With the results we demonstrated here, the more detailed information for captive sika deer intestinal microbiome in future could provide us with ideas that protect wild sika deer in Northeast China, such as the change of vegetation types by artificial cultivation with the analysis of energy metabolism pathway or establish the specialized nature reserve for wild sika deer in these areas. Notably, the National Park for Amur Tiger and Amur Leopard that will be built in 2020 in China is also the essentially key habitat for wild sika deer, which could protect this precious species effectively. It is true that the progress of next-generation sequencing technology impels our comprehension to gut microbiota of animals, but there still are shortcomings of it and bacterial communities with unknown functions need to be identified in further investigations as the advancement of technology and arithmetic.

Our study for sika deer characterized the fundamental composition and structure of gut microbiota from feces using high-throughput sequencing technology, and revealed the significant differences in gut microbiota at various levels between wild and captive sika deer. We inferred that the higher bacterial species diversity and relative abundance in captive sika deer were mainly because of the high-fiber and sufficient food source during winter in deer farm. And for wild sika deer, *Tenericutes* is a dominant phylum that ranked before *Proteobacteria* and *Actinobacteria*, which may indicate its essential role in regulating the gut ecosystem homeostasis and health for host. Hence, *Tenericutes* deserves to be taken seriously in wild sika deer for its specific functions and molecular mechanism in gut microbiota. Also, the metabolic pathway of these bacterial species is the following direction of study by metagenome to explore the deeper mechanism. Furthermore, the special artificial environment of deer farm and variations in gut microbiota during seasons also should be considered as vital factors which may provide insights for animal feeding and protection for wild animals.

## Additional files

**Additional file 1: Figure S1.** ANOSIM analysis for discrepancy of fecal bacterial community between wild and captive sika deer. The difference between groups here was greater than it within each group because the R value was less than 0, and the P value showed the significance level.

**Additional file 2: Figure S2.** T test bar plot for analysis of species discrepancies between two groups at phylum (a), family (b) and genus (c) level. Each bar on the left figure represents the means of relative abundance of species that showed significant difference between wild and captive group. The P-value of t-test and the difference (the center of a circle) of means with lower and upper confidence interval limits were demonstrated in the right figure.

## Abbreviations

PCR: polymerase chain reaction
rRNA: ribosomal ribonucleic acid
OTU: operational taxonomic unit
ANOSIM: analysis of similarities
PCA: principal component analysis
PCoA: principal co-ordinate analysis
NMDS: non-metric multidimensional scaling
UPGMA: unweighted pair-group method with arithmetic means
LEfse: linear discriminant analysis coupled with effect size
