# Correction to: Comparison of the gut microbiota composition between wild and captive sika deer (*Cervus nippon hortulorum*) from feces by high-throughput sequencing

**DOI:** 10.1186/s13568-018-0535-1

**Published:** 2018-02-05

**Authors:** Yu Guan, Haitao Yang, Siyu Han, Limin Feng, Tianming Wang, Jianping Ge

**Affiliations:** Ministry of Education Key Laboratory for Biodiversity Science, and Engineering and College of Life Sciences, Beijing Normal University, No. 19, Xinjiekouwai Street, Haidian District, Beijing, 100875 People’s Republic of China

## Correction to: AMB Expr (2017) 7:212 10.1186/s13568-017-0517-8

The authors regret the following errors occurred in the original publication of the article (Guan et al. 2017). The corrected texts have been presented with this erratum.

In Abstract section, the sentence should read, “Here, we characterized the gastrointestinal bacterial communities of wild (7 samples) and captive (12 samples) sika deer from feces, and compared their gut microbiota by analyzing the V4 region of 16S rRNA gene using high-throughput sequencing technology on the Illumina Hiseq platform”.

In the section titled, “16S rRNA gene PCR and sequencing”, the first paragraph should read, “16S rRNA gene was amplified using the 16S universal amplicon PCR primers: F515 (5′-CACGGTCGKCGGCGCCATT-3′) and R806 (5′-GGACTACHVGGGTWTCTAAT-3′), and V4 region of 16S rRNA gene were our final target fragments for sequencing. A total final volume of 50 μl mixture for polymerase chain reaction: 6 μl of template fecal DNA, 25 μl of 2× Taq PCR Master Mix (0.1 U/μl), 2 μl of each primer (10 μM) and 15 μl ddH_2_O to complement the reaction system. Then DNA was amplified using the conditions below: 3 min at 94 °C for initial denaturation, then followed 28 cycles of 94 °C for 30 s, 53 °C for 40 s and 72 °C for 1 min. Finally, followed by an extension step of 72 °C for 5 min”.

These corrections do not alter any conclusions or results of our study.
