# Supplementary figures and images for: Comparison of the gut microbiota composition between wild and captive sika deer (Cervus nippon hortulorum) from feces by high-throughput sequencing
Source: AMB Express. 2017 Nov 23;7:212. doi: 10.1186/s13568-017-0517-8 (PMC5700909; doi:10.1186/s13568-017-0517-8)

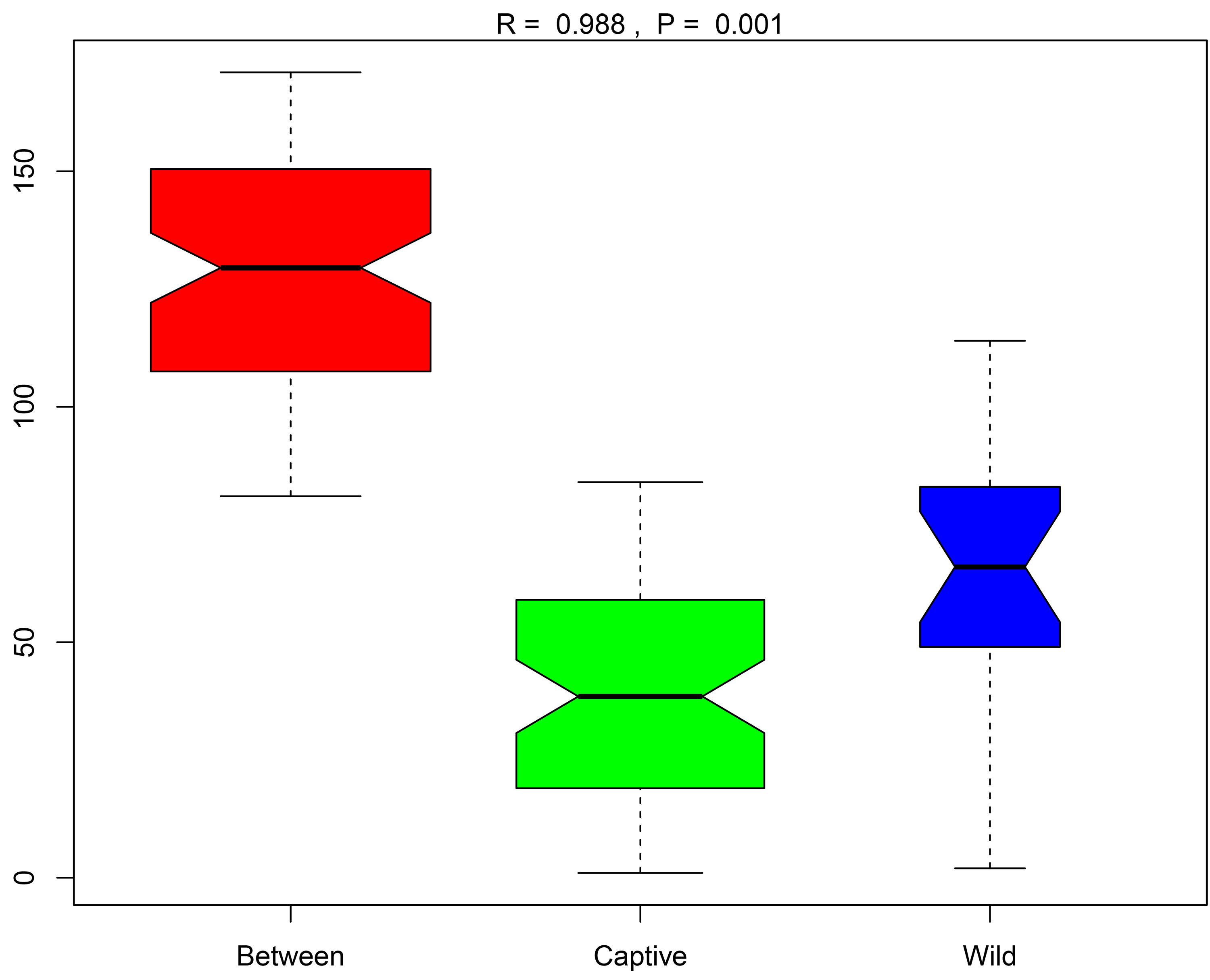

Supplement: Supplementary file 1 — Additional file 1: Figure S1. ANOSIM analysis for discrepancy of fecal bacterial community between wild and captive sika deer. The difference between groups here was greater than it within each group because the R value was less than 0, and the P value showed the significance level. [file 13568_2017_517_MOESM1_ESM.tif]

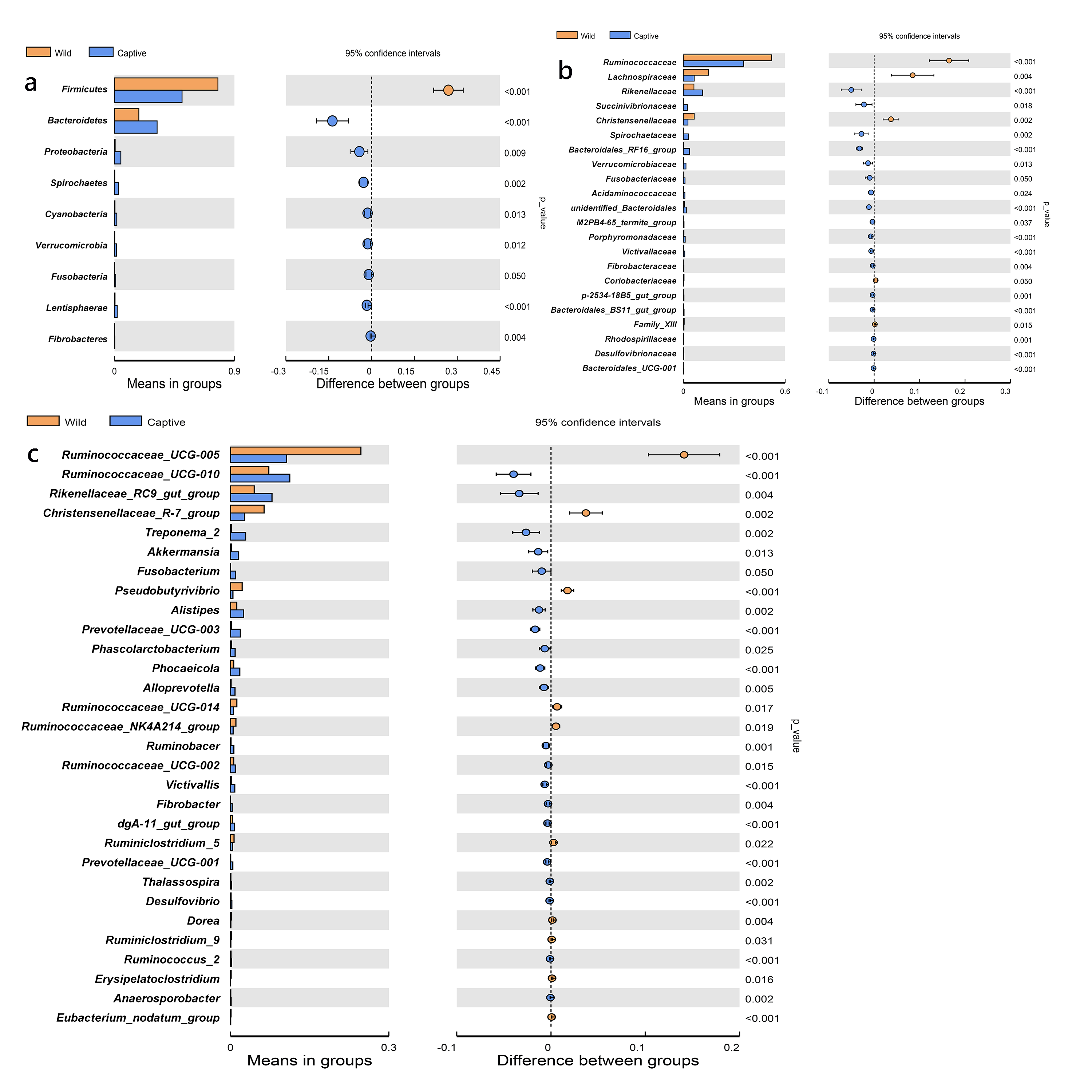

Supplement: Supplementary file 2 — Additional file 2: Figure S2. T test bar plot for analysis of species discrepancies between two groups at phylum (a), family (b) and genus (c) level. Each bar on the left figure represents the means of relative abundance of species that showed significant difference between wild and captive group. The P-value of t-test and the difference (the center of a circle) of means with lower and upper confidence interval limits were demonstrated in the right figure. [file 13568_2017_517_MOESM2_ESM.tif]
